# Prevalence and characterization of ESBL-producing diarrheagenic *Escherichia coli* from the poultry industry in Shanghai, China

**DOI:** 10.3389/fmicb.2025.1573614

**Published:** 2025-06-30

**Authors:** Fengxia Que, Jiachun Yuan, Xinyue Xu, Chunfu Liu, Yunyan Yang, Yulong Ye

**Affiliations:** Jinshan District Center for Disease Control and Prevention (Jinshan District Health Supervision Institute), Shanghai, China

**Keywords:** ARGS, ESBL-producing diarrheagenic *Escherichia coli*, WGS, MDR, SNP

## Abstract

**Objective:**

Antibiotic resistance genes (ARGs) in bacteria are highly prevalent in the poultry industry. These genes can transfer not only within the same species but also across different species, posing significant threats to both the poultry industry and human health. However, limited research has been conducted on the prevalence and transmission links between extended-spectrum beta-lactamase (ESBL)-producing diarrheagenic *Escherichia coli* (*E. coli*) in chickens and their living environments in Shanghai’s poultry industry.

**Methods:**

In this study, 600 samples were collected from the cloacal swabs of healthy chickens and from the environments of poultry farms. These samples included feces, troughs, water containers, and soils. Antimicrobial susceptibility testing and whole-genome sequencing (WGS) were employed to characterize ESBL-producing diarrheagenic *E. coli*.

**Results:**

The results indicated a detection rate of *E. coli* at 15.67%, with the isolates exhibiting high resistance to the tested antibiotics, particularly ampicillin (AMP),nalidixic acid (NAL), chloramphenicol (CHL), and tetracycline (TET). Notably, 95.45% of the isolates were multidrug-resistant (MDR). Among these isolates, 20.45% were ESBL-producing *E. coli*, which exhibited higher resistance to first- through fourth-generation cephalosporins, such as cefazolin (CFZ),cefuroxime (CXM), and cefotaxime (CTX). These ESBL-producing *E. coli* also carried a significant number of ARGs, with *bla_TEM-1B_* (55.56%) and *bla_CTX-M-55_* (38.89%) being the most prevalent on the farm. The dominant sequence type (ST) was ST1286, the prevalent serotype was O16: H32, and the dominant CH type was FumC174/FimH23. Isolates that shared the same ST types clustered together and exhibited the same serotypes and CH types.

**Conclusion:**

The findings from this study reveal that ARGs are widely distributed among ESBL-producing *E. coli* strains. STs with the same serotypes and FumC-FimH (CH) types showed high genetic relatedness in single-nucleotide polymorphism (SNP)-based phylogenetic analysis, highlighting the need for enhanced surveillance to prevent further transmission.

## 1 Introduction

*Escherichia coli* (*E. coli*) is a ubiquitous bacterium found in the guts of humans and animals, as well as in various environmental sources. In most cases, it acts as an opportunistic pathogen that does not cause severe diseases. However, there are concerns about the spread and infection risks associated with specific diarrheagenic strains of *E. coli*. Based on their virulence and phenotypic characteristics, several pathogenic subtypes of *E. coli* have been identified. These subtypes include enterotoxigenic *E.coli* (ETEC), enteroinvasive *E.coli* (EIEC), enterohemorrhagic *E.coli* (EHEC), enteropathogenic *E.coli* (EPEC), and enteroaggregative *E. coli* (EAEC). In addition, diffusely adherent *E. coli* (DAEC) and adherent-invasive *E. coli* (AIEC) have also been identified (Alizade et al., 2019). These pathogenic *E. coli* strains can cause various diseases in humans, such as diarrhea, enteritis, septicemia, and other infections (Ramos et al., 2020).

Antibiotic resistance has become a major global health crisis due to the widespread emergence of resistant pathogens (Cižman and Plankar Srovin, 2018). It is impossible to overlook the problem of antibiotic resistance in animal consumption, given that animal consumption in 2013 accounted for 52% of total antibiotic use in China (Zhang et al., 2015). Bacteria such as *E. coli* act as reservoirs and vectors capable of transmitting resistance genes to humans through food consumption or environmental exposure (Schaefer et al., 2011). Therefore, it is crucial to implement rigorous surveillance of antibiotic-resistant bacteria and enforce measures to mitigate their transmission.

Globally, over 1.5 billion people were found to be infected with extended-spectrum β-lactamase (ESBL)-producing *E. coli*, with the highest prevalence in developing countries (Woerther et al., 2013). ESBLs, including TEM, SHV, and CTX-M enzyme families, are bacterial enzymes capable of hydrolyzing and inactivating β-lactam antibiotics (Smet et al., 2010). These resistance determinants are encoded by genes located on plasmids or chromosomes of Enterobacteriaceae (Bradford, 2001). The World Health Organization (WHO) classifies ESBL-producing Enterobacteriaceae as critical priority pathogens due to their significant role in complicating clinical treatments and increasing patient morbidity rates (Maslikowska et al., 2016). Additionally, the dissemination of ESBL-producing *E. coli* in animals exacerbates this public health challenge. Livestocks, which are important nutritional sources for humans, have been identified as significant reservoirs of ESBL-producing *E. coli* (Yang et al., 2022). Furthermore, the rapid emergence of ESBLs in *E. coli* may indicate the development of multidrug resistance (MDR) in livestocks (Wu et al., 2018).

Antibiotic resistance genes (ARGs) in bacteria are highly prevalent in the poultry industry and demonstrate remarkable transferability, allowing them to spread not only within bacterial species but also across different species (Poole et al., 2017; Davies, 1994). This phenomenon poses significant threats to both poultry production systems and public health (Choi et al., 2021). In addition to livestocks, environments contaminated with animal feces also contain abundant ARGs in bacteria (Peng et al., 2021). Notably, studies have identified higher concentrations of bacterial ARGs in chicken feces compared to other livestock species (Qian et al., 2018).

Whole-genome sequencing (WGS) is a powerful tool for analyzing bacterial genetic traits, including antibiotic resistance genes and multilocus sequence typing (MLST). MLST classifies bacterial strains based on several housekeeping genes (Ramadan et al., 2020). In addition, CH typing, a method specifically adapted for *E. coli*, was developed to study the clonal diversity of sub-sequence types (STs). This approach utilizes *fumC*, which is one of the household genes used in MLST, and *fimH*, which is an internal fragment of the type 1 fimbrial adhesin-encoding gene, demonstrating superior reliability and feasibility in assessing clonality (Johnson and Russo, 2018; Vautrin et al., 2023).

In recent years, studies on *E. coli* in livestock farms have primarily focused on regions in China outside of Shanghai (Luo et al., 2023; Afayibo et al., 2022), with limited analysis of the prevalence and epidemiological links between ESBL-producing diarrheagenic *E. coli* strains from chickens and their surrounding environments in the poultry industry. Therefore, this study aimed to characterize the genetic features (e.g., resistance profiles, ARGs, MLST, serotypes, and CH types) of ESBL-producing pathogenic *E. coli* and provide long-term insights for improving poultry farming practices and infection prevention strategies.

## 2 Materials and methods

### 2.1 Sample collection

The Jinshan district, located in the southwest region of Shanghai, had nine chicken farms, all operated by breeding cooperatives. Two of these farms (A and B) were included in this study. Farm A had more than 10 chicken sheds, while Farm B had 5–6 sheds. Each shed housed over 1,000 chickens. Each of these two farms had three farming cycles annually, with a one-month break between cycles. The chickens had a feeding cycle of 90 days.

Samples were collected in 2021 from the cloacal swabs of healthy chickens and the surrounding environments of the two poultry farms —including troughs, water containers, soils, and feces—over the course of four quarters. A total of 300 samples were obtained from each breeding farm, resulting in an overall total of 600 samples. Each sample was collected from chicks approximately 1 month old. It is important to note that the breeding cycles of the chicken coops were not synchronized. For example, coops one through three were in the rest period, while coops four through seven were in the breeding cycle. As a result, we were able to collect samples during each quarterly sampling session. After collection, all samples were delivered to the laboratory for examination within 2 h.

### 2.2 Strains and culture conditions

The cloacal swab samples were placed in a nutrient broth enrichment medium and incubated for 24 h at 37°C, in accordance with the Hygienic Standard WS 271–2007 (Ministry of Health, People’s Republic of China, 2007). After inoculating the cultures onto MacConkey agar, they were incubated for an additional 24 h at 37°C. Matrix-assisted laser desorption/ionization time-of-flight mass spectrometry (MALDI-TOF MS) (Autobio, Zhengzhou, China) was used to select and identify questionable colonies. PCR typing of *E. coli* was performed using an ABI 7500 system (USA) to verify diarrheagenic *E. coli* strains, following the manufacturer’s instructions. A total of 94 strains of diarrheagenic *E. coli* were identified and preserved.

### 2.3 Antimicrobial susceptibility testing

According to the micro broth dilution procedure, 23 antibiotics included in the kit were used (Fosun Diagnostics, China). *E.coli* (ATCC25922) was used as the quality-control strain. Ceftazidime/clavulanic acid and cefotaxime/clavulanic acid were used for the phenotypic determination of extended-spectrum β-lactamase (ESBL)-producing diarrheagenic *E. coli*, following the CLSI M100 30th edition criteria—that is, a decrease of ≥ 3 dilutions in the MIC value for either of the two drugs with or without clavulanic acid. Multidrug-resistant (MDR) bacteria were defined as those that exhibited resistance to at least one agent in three or more antimicrobial groups (Okasha et al., 2023).

### 2.4 Whole-genome sequencing (WGS)

A magnetic bead-based method was employed to extract bacterial genomic DNA (gDNA) (BioPerfectus, Jiangsu, China). Its purity and concentration were assessed by nucleic acid and protein fluorescence quantification (Thermo Fisher Scientific, USA). The library was constructed using the MGIEasy Enzyme Digestion DNA Library Preparation Reagent Set, according to the manufacturer’s protocol (MGI, Shenzhen, China). Following fragmentation, repair, and adapter ligation, the gDNA was purified and amplified using PCR. The PCR products were directly used for rapid DNA nanoball (DNB) preparation with the DNBSEQ OneStep DNB Preparation Kit (MGI, Shenzhen, China). Paired-end 150-bp reads were generated using the DNBSEQ platform (MGI, Shenzhen, China). The obtained sequences were subjected to QC using MGAP v 2.0.0 to filter out low-quality or substandard reads, ensuring that only high-quality clean reads were retained for genome assembly. The website https://pubmlst.org/ was used to determine the MLST type. The Center for Genomic Epidemiology^1^ was consulted for the analysis of the isolates for FumC-FimH (CH) types, serotypes, and ARGs.

### 2.5 Single-nucleotide polymorphism (SNP)

One of the samples was used as a reference, and single-nucleotide polymorphism (SNP) sites were identified among the samples using Snippy 4.6.0 software. A SNP-based phylogenetic tree was generated using FastTree 2.1.7 with neighbor-joining clustering, based on the multiple sequence alignment of the SNP sites across the entire genomes.

### 2.6 Statistical analysis

Data analysis was performed using SPSS 19.0. A chi-squared test was performed to compare differences in the detection rates of *E.coli* between the environmental samples and cloacal swabs from healthy chickens, across the four sampling quarters, and between the two farms. In addition, it was also used to compare differences in antibiotic resistance between strains that produced ESBL and strains that did not. In all statistical analyses, a *p*-value of < 0.05 was considered indicative of a significant difference.

## 3 Results

### 3.1 Total detection

**Figure 1:**
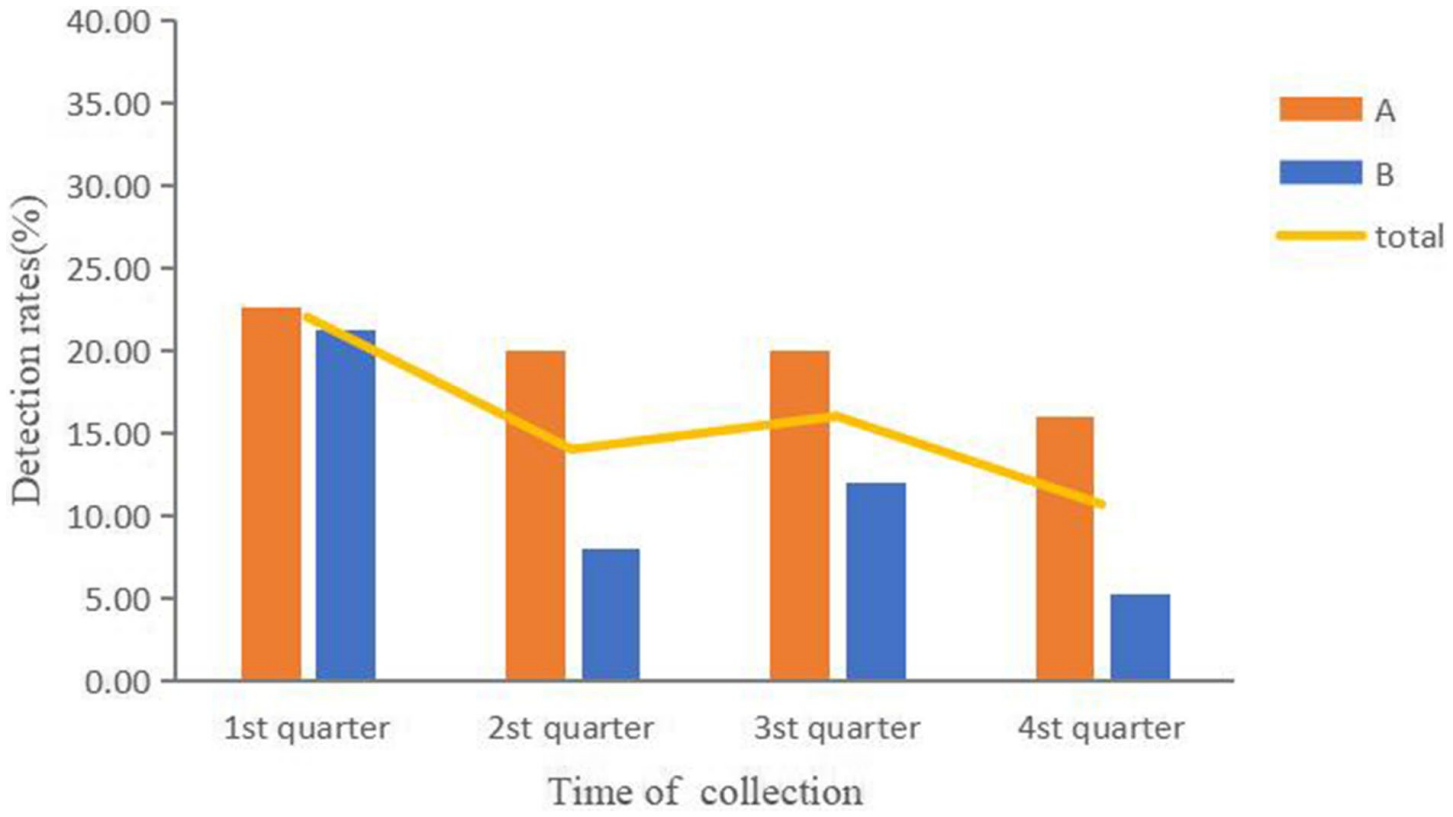
Detection rates of diarrheagenic *E. coli* in the two poultry farms.

Of the samples tested, 15.67% (94/600) were found to be positive for diarrheagenic *E. coli*. In the cloacal samples from healthy chickens, the detection rate was significantly higher at 28.75% (69/240) compared to a rate of 6.94% (25/360) in environmental samples (*χ^2^* = 51.82, *p* < 0.001). The findings of the PCR test showed that 93 strains were Enteroaggregative *E. coli*, whereas 1 strain was Enteropathogenic *E. coli*. As illustrated in Figure 1, the detection rate of diarrheagenic *E. coli* was highest in the first quarter (22.0%) and lowest in the fourth quarter (10.67%). However, there was no significant difference in detection rates between the four quarters (*χ^2^* = 7.72, *p > 0.05*). Farm A had a higher detection rate (19.67%, 59/300) than Farm B (11.67%, 35/300) (*χ^2^* = 7.27, *p* < 0.01).

### 3.2 Antibiotic resistance profiles

**Figure 2:**
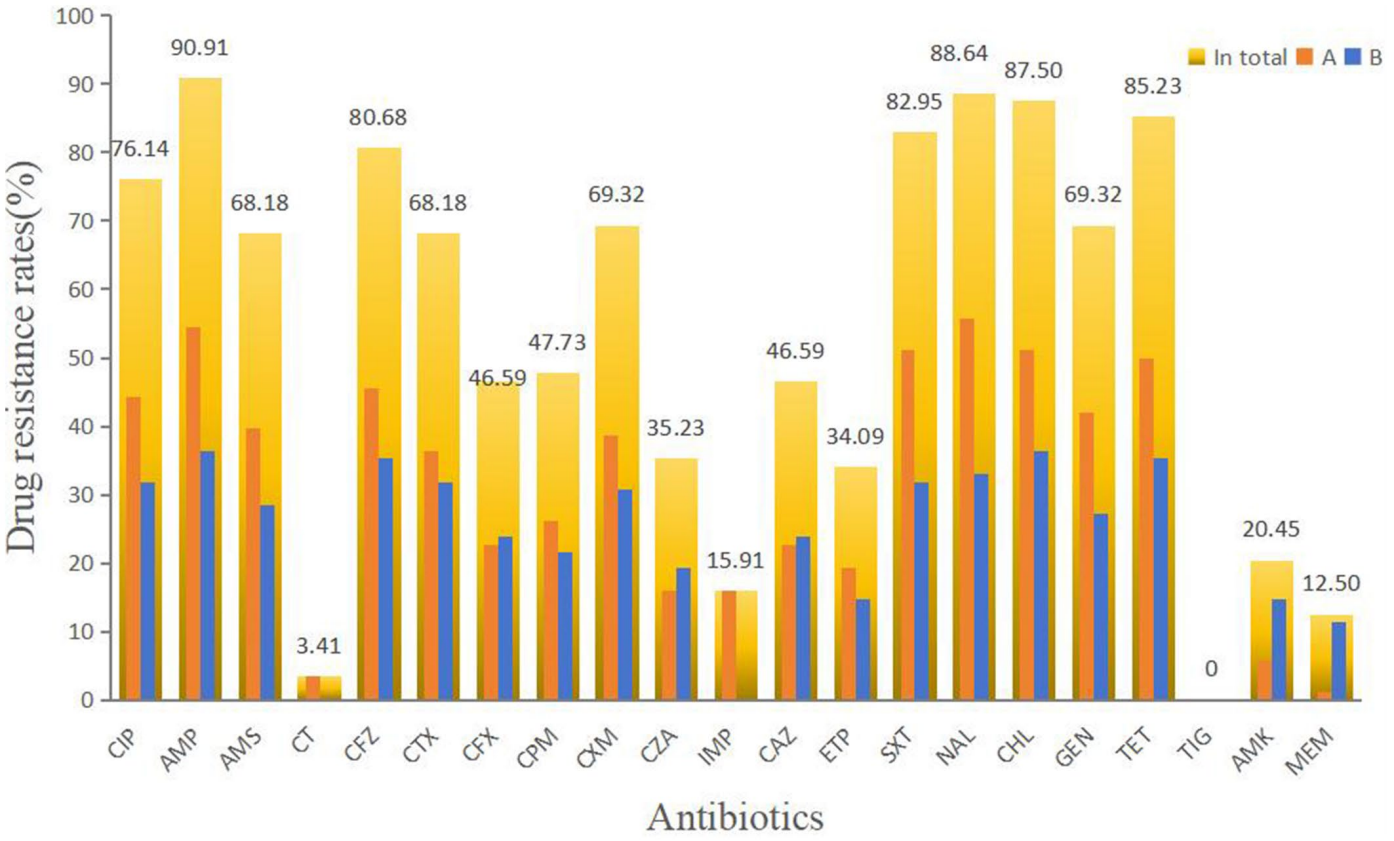
Antimicrobial susceptibility results of diarrheagenic *E. coli*. CIP, ciprofloxacin; AMP, ampicillin; AMS, ampicillin/sulbactam; CT, polymyxinE; CFZ, cefazolin; CTX, cefotaxime; CFX, cefoxitin; CPM, cefepime; CXM, cefuroxime; CZA, ceftazidime/avibactam; IMP, imipenem; CAZ, ceftazidime; ETP, ertapenem; SXT, trimethoprim-sulfamethoxazole; NAL, nalidixic acid; CHL, chloramphenicol; GEN, gentamicin; TET, tetracycline; TIG, tigecycline; AMK, amikacin; and MEM, meropenem.

Out of 94 isolates, 88 were successfully resuscitated. Among these, 87 (98.86%) exhibited drug resistance. Resistance to ampicillin (AMP) was observed in 90.91% of the isolates, followed by resistance to nalidixic acid (NAL) at 88.64%, chloramphenicol (CHL) at 87.50%, tetracycline (TET) at 85.23%, trimethoprim-sulfamethoxazole (SXT) at 82.95%, and cefazolin (CFZ) at 80.68%. Over 50% of the isolates showed antibiotic resistance to ciprofloxacin (CIP), ampicillin/sulbactam (AMS), cefotaxime (CTX), cefuroxime (CXM), and gentamicin (GEN). Resistance to polymyxin E (CT), imipenem (IMP), amikacin (AMK), and meropenem (MEM) was 20% or less. No isolates showed resistance to TIG. Only one isolate exhibited resistance to IMP. All isolates resistant to CT and IMP were from Farm A (Figure 2). The isolates in Farm B were primarily responsible for the high rate of MEM resistance. Overall, 95.45% of the isolates (84/88) were classified as MDR, with all isolates from Farm B exhibiting MDR profiles. Among 54 resistance profiles, 79 isolates exhibited resistance to at least 5 antibiotics. CIP-AMP-AMS-CFZ-CTX-CFX-CPM-CXM-CZA-CAZ-ETP-SXT-NAL-CHL-GEN-TET and CIP-AMP-AMS-CFZ-CTX-CFX-CPM-CXM-CZA-IMP-CAZ-ETP-SXT-NAL-CHL-GEN-TET-MEM were the main resistance profiles. Six strains were included in each of these resistance profiles.

### 3.3 Antibiotic resistance profiles of ESBL-producing diarrheagenic *Escherichia coli*

**Figure 3:**
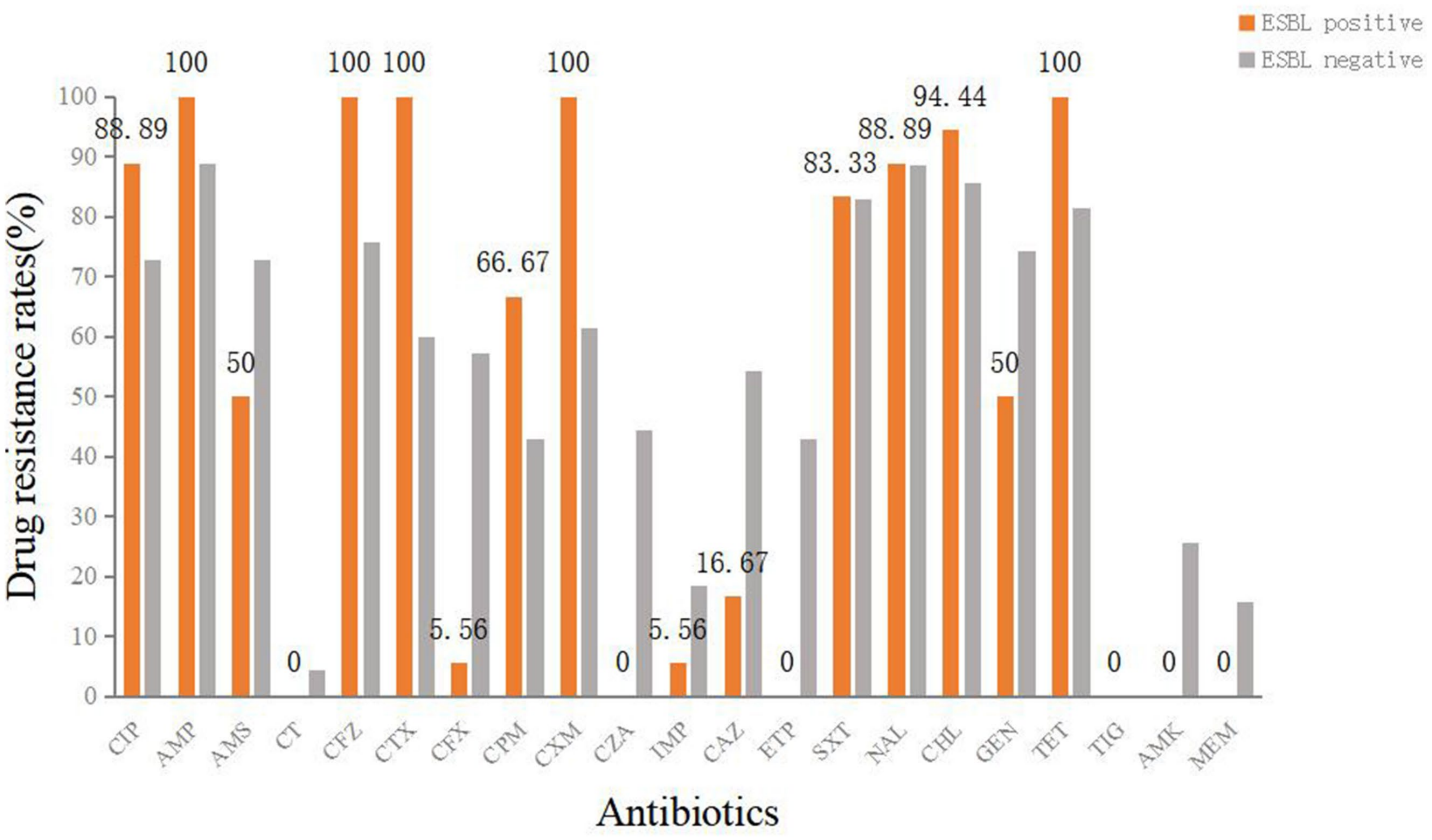
Antimicrobial susceptibility results of ESBL-producing diarrheagenic *E. coli* and non-ESBL-producing diarrheagenic *E. coli.*

Of the 88 isolates, 18 (20.45%) were ESBL-producing diarrheagenic *E. coli*. The remaining isolates were obtained from the environmental samples, while 15 were from the cloacal samples. The ESBL-producing strains showed no resistance to CT, CZA, ETP, TIG, MEM, or AMK. However, they were 100% resistant to AMP, CFZ, CTX, CXM, and TET. Significant differences between the ESBL-producing and non-ESBL-producing strains were found in CFZ (*χ^2^* = 3.97, *p* < 0.05), CTX (*χ^2^* = 10.56, *p* < 0.01), CXM (*χ^2^* = 10.02, *p* < 0.01), CZA (*χ^2^* = 12.31, *p* < 0.001), CAZ (*χ^2^* = 8.14, *p* < 0.01), ETP (*χ^2^* = 11.70, *p* < 0.001), GEN (*χ^2^* = 3.97, *p* < 0.05), and AMK (*χ^2^* = 4.34, *p* < 0.05) (Figure 3).

### 3.4 Screening for ARGs in ESBL-producing diarrheagenic *Escherichia coli*

A total of 11 families of ARGs were identified, including those conferring resistance to aminoglycosides, β-lactams, methotrexates, tetracyclines, chloramphenicols, sulphonamides, quinolones, rifamycins, lincoamides, macrolides, and polyphosphates. Among these, *tet(A)* exhibited the highest detection rate at 77.78%, followed by *floR* at 72.22%, *sul2* at 61.11%, and *bla_TEM-1B_* at 55.56%. Other ARGs were detected at varying rates. Notably, each strain contained at least four ARGs, representing at least three distinct classes.

**Figure 4:**
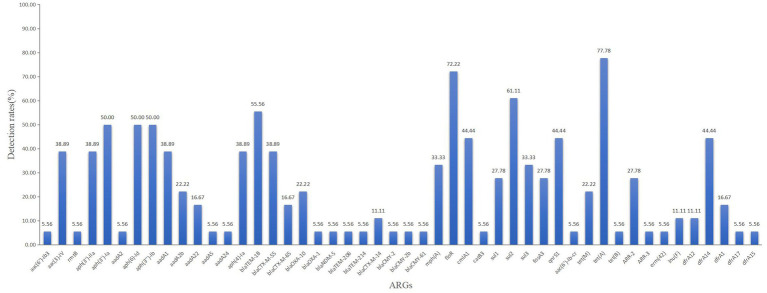
ARGs in ESBL-producing diarrheagenic *E. coli.*

A total of 15 isolates (83.33%) harbored β-lactamase genes. The *bla_CTX-M_* and *bla_TEM_* genes were detected in 61.11% (11/18)and 55.56%(10/18) of the isolates, followed by *bla_OXA_* (27.78%). Particularly, *bla_TEM-1B_* was the most prevalent variant (55.56%), followed by *bla_CTX-M-55_* (38.89%) and *bla_OXA-10_* (22.22%). The genes *bla_CMY-2_*, *bla_CMY-2b_*, and *bla_CMY-61_*, which encode AmpC-β-lactamases, were all detected in one isolate. Another isolate carried NDM-5, a metallo-β-lactamase that can hydrolyze the majority of carbapenems (Figure 4).

Two or more β-lactamase genes were detected in 11 of the 18 isolates. *Bla_CTX-M-55_* and *bla_TEM-1B_* (two isolates), *bla_CTX-M-55_* and *bla_OXA-10_* (two isolates), *bla_CTX-M-65_* and *bla_TEM-1B_* (one isolate), and *bla_CTX-M-14_* and *bla_TEM-1B_* (one isolate) were the genes that coexisted. One isolate harbored three coexisting genes: *bla_OXA-10_*, *bla_CTX-M-65_*, and *bla_TEM-1B_*. Another isolate carried *bla_CMY-2b_*, *bla_CMY-61_*, and *bla_CMY-2_*, while a third isolate carried *bla_CTX-M-65_*, *bla_TEM-1B_*, and *bla_OXA-1_*. Four β-lactamase genes were present in two separate isolates: one isolate carried *bla_TEM-214_*, *bla_TEM-206_*, *bla_TEM-1B_*, and *bla_NDM-5_*, while the other carried *bla_CTX-M-14_*, *bla_OXA-10_*, *bla_CTX-M-55_*, and *bla_TEM-1B_* (Table 1).

**Table 1:** Carriage of β-lactamase genes in the 18 isolates.

Strains	Industry farm	β-lactamases	ARGs
2,021,024	B	CTX-M-55	5
2,021,029	B	TEM-1B	11
2,021,030	B	CTX-M-55、TEM-1B	11
2,021,031	B	CTX-M-55、OXA-10	14
2,021,032	B	TEM-214、TEM-206、TEM-1B、NDM-5	15
2,021,033	B	CTX-M-65、TEM-1B	14
2,021,037	A	TEM-1B、CTX-M-55	14
2,021,039	A	CTX-M-14、OXA-10、CTX-M-55、TEM-1B	21
2,021,045	A	OXA-10、CTX-M-55、	13
2,021,050	A	TEM-1B	7
2,021,053	A	CTX-M-14、TEM-1B	13
2,021,056	A	OXA-10、CTX-M-65、TEM-1B	19
2,021,057	A	CMY-2b、CMY-61、CMY-2	5
2,021,066	A	–	4
2,021,069	A	CTX-M-55	10
2,021,076	A	–	4
2,021,084	A	–	10
2,021,091	A	CTX-M-65、TEM-1B、OXA-1	16

### 3.5 Typing and phylogenetic tree

**Figure 5:**
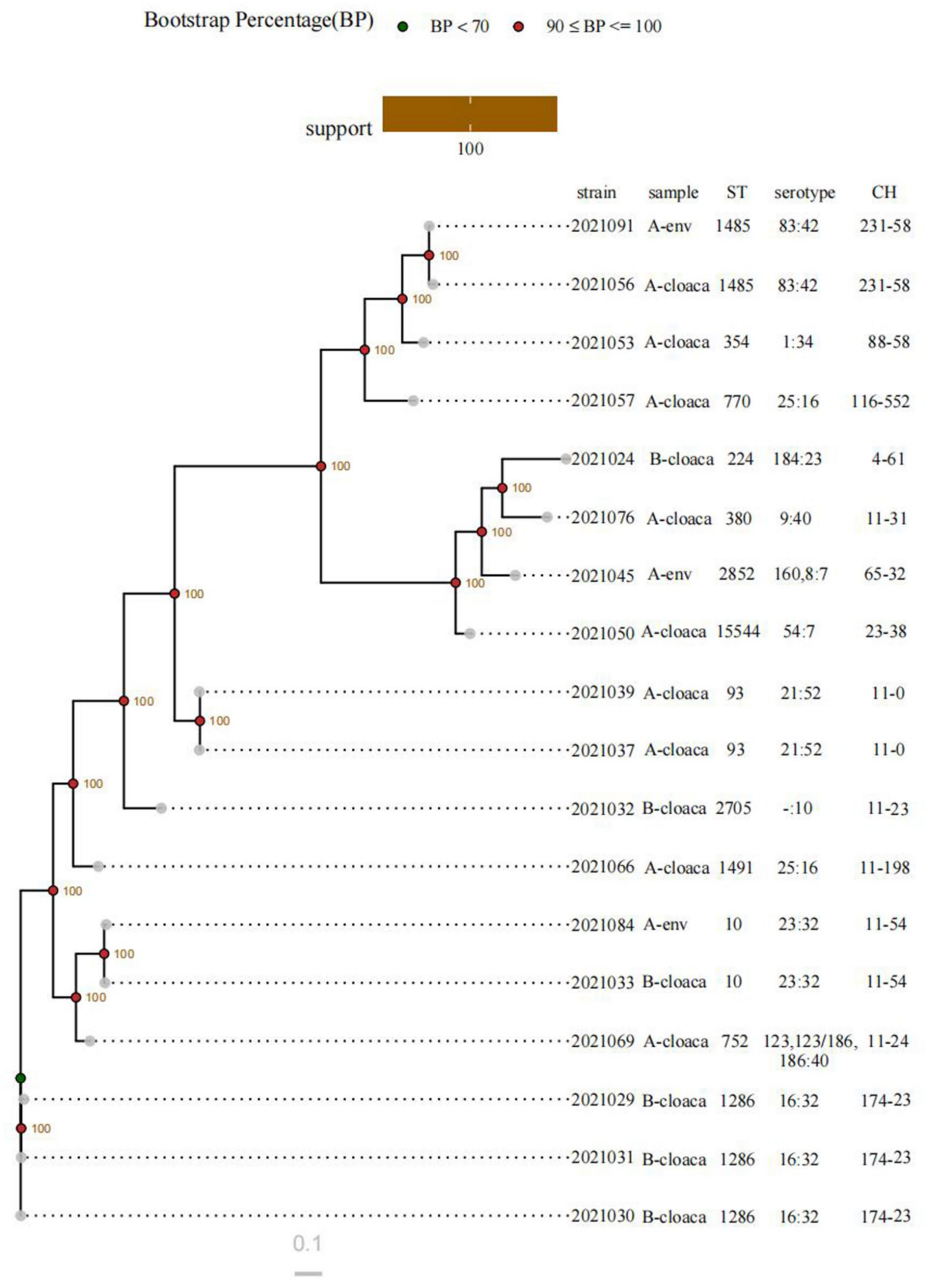
Phylogenetic tree of ESBL-producing diarrheagenic *E. coli* based on genome-wide SNP analysis. env indicates environmental samples. - indicates not detected.

The MLST technique was used to identify a total of 13 STs. The most common ST was ST1286, found in 16.67% of the isolates (three isolates). This was followed by ST93, ST1485, and ST10, each of which was found in two isolates. A total of 12 serotypes were identified, with O16: H32 (three isolates) being the most common. The serotypes O83: H42, O21: H52, O25: H16, and O23: H32 ranked next, each represented by two isolates. A total of 13 CH types were observed, with FumC174/FimH23 (three isolates) being the most frequent. FumC11/FimH54, FumC11/FimH0, and FumC231/FimH58 were also detected, each in two isolates. C11 (eight isolates) and H23 (four isolates) were the predominant *fumC* and *fimH* types, respectively. In this study, the isolates sharing the same STs exhibited identical serotypes and CH types, while others demonstrated significant heterogeneity (Figure 5).

Based on an SNP-based phylogenetic tree, the strains were divided into two major clusters. One cluster consisted of three isolates collected from the cloacal swabs of healthy chickens on Farm B (all ST1286). The remaining isolates formed the second cluster. The cluster comprised three closely related subclusters: ST93, ST10, and ST1485, with each subcluster containing two isolates. ST93 isolates were exclusively obtained from the cloacal samples of healthy chickens on Farm A. ST1485 isolates originated from one cloacal sample and one environmental sample, both from Farm A. 2,021,033 and 2,021,084 strains (both assigned to ST10) were isolated from a cloacal sample obtained from Farm B and an environmental sample from Farm A, respectively.

## 4 Discussion

In this study, we investigated the prevalence, resistance profiles, and genetic characteristics of diarrheagenic *E. coli* in Shanghai, China. The occurrence rate of diarrheagenic *E. coli* was found to be 15.67%. Diarrheagenic *E. coli is* a key target in pathogen-monitoring programs for livestock and poultry products in various countries (Mohamed et al., 2018; Wang et al., 2021). These bacteria can cause diarrhea, and their primary route of transmission is through contaminated food (Aijuka and Buys, 2019). Consistent with the natural intestinal colonization of *E. coli*, the detection rate was significantly higher in cloacal swabs compared to environmental samples. This finding was also confirmed by Guo et al. (2021), who compared the different sources of *E. coli*. However, it should not be overlooked that environmental samples, such as water containers, feces, and soil, may serve as vectors of pathogenic bacteria and ARGs (Mandal et al., 2022). Improper management of fecal waste and water troughs may lead to environmental contamination (Jechalke et al., 2014; Fučík et al., 2024). We suggest that factors such as farm size, flock density, and disinfection protocols may account for the observed differences in detection rates between the two farms studied. Although quarterly sampling revealed no significant seasonal variation in the detection rates, we observed a significant difference in bacterial isolation between Q1 and Q4. This difference may be attributed to more favorable temperatures for bacterial growth during these periods.

The isolated *E. coli* exhibited high resistance to the tested drugs. The high resistance rates to AMP, NAL, CHL, and TET are consistent with the findings reported by Zou et al. (2021). According to the list of banned veterinary medications, CHL is prohibited in food animal breeding. Nevertheless, up to 87.50% of the isolates in this study exhibited resistance to CHL. It may be necessary for the relevant authorities to conduct additional monitoring to determine whether CHL has been used in feeding practices and whether it is present in chicken products. Antibiotics have been heavily used in animal feed for decades (Collignon and Voss, 2015). The resistance rates to first- through fourth-generation cephalosporins exceeded 40%, with the highest resistance observed for CFZ at 80.68%. In addition, the isolates showed varied resistance to carbapenem antibiotics (12.50–34.09%). Colistin is considered a last-resort option for treating MDR Enterobacteriaceae (Sato et al., 2018). In this study, three CT-resistant isolates from Farm A were found to be non-ESBL-producers. It is still unclear whether the remaining non-ESBL strains of *E. coli* carry the *mcr* gene because we only sequenced the genomes of ESBL-producing bacteria.

We observed that the level of antibiotic resistance varied between the two farms. This variation is likely due to slight differences in antibiotic usage, as indicated by the fact that the presence of IMP-resistant isolates were exclusively found on Farm A, while MEM-resistant isolates predominantly originated from Farm B.

A broad spectrum of resistance to up to 18 antibiotics was observed, indicating a serious MDR level. ESBL-producing *E. coli* may serve as a useful marker for the surveillance of MDR *E. coli* in the food animal breeding industry (Wu et al., 2018). The percentage of ESBL-producing *E.coli* was 20.45%, which is much lower than the 78.2% reported in another study (Liu et al., 2022). This discrepancy is likely due to the differences in sample types and the geographical areas covered in this research. The data also revealed differences in resistance to certain antibiotics—such as cephalosporins from the first to the third generations—between the isolates that produced ESBL and those that did not. The production of ESBLs was the primary cause of cephalosporin resistance.

The production and dissemination of β-lactamases significantly reduce the effectiveness of β-lactam antibiotics, posing a serious threat to human health and animal breeding. Among the 18 isolates, 11 harbored more than two β-lactamase genes. In recent years, CTX-M has become increasingly prevalent, surpassing TEM, which was previously one of the most common ESBLs worldwide (de Carvalho et al., 2020). In addition, the CTX-M family is the primary mechanism by which *E. coli* develops resistance to third-generation cephalosporins (Bush and Bradford, 2020). Both *bla_CTX-M_* and *bla_TEM_* were prevalent in the study, accounting for 61.11% and 55.56% of the ESBL-producing *E. coli*. The prevalence of *bla_CTX-M_* was higher than the 38.8% reported by Zhao et al. (2022) but lower than the 99.6 and 92.7% reported by Kiratisin et al. (2008), and Wu et al. (2018), respectively. It is believed that the prevalence of *bla_CTX-M_* in *E. coli* varies among different animals and geographical areas, potentially resulting from the antibiotics used and the surrounding conditions. The study identified *bla_CTX-M-55_* (38.89%) and *bla_CTX-M-65_* (16.67%) as the predominant *bla_CTX-M_* variants. Given that the most common quinolone resistance gene, *qnrS1*, accounted for 44.44%, and *aac (6′)-Ib-cr* accounted for 5.56%, the rates for quinolone resistance genes were significantly lower than that for CIP phenotypic resistance (88.89%). It is hypothesized that additional processes, such as antibiotic efflux, may contribute to quinolone resistance (Jian et al., 2021).

This investigation identified a variety of ARGs in ESBL-producing *E. coli*. Each isolate harbored genes from at least three classes and four kinds of ARGs. The *tet(A)* gene exhibited the highest prevalence at 77.78%, followed by *floR* (72.22%) and *sul2* (61.11%), all of which were shown to be consistent with the observed drug-resistant phenotypes. The MDR phenomenon further supports the fact that β-lactamases, in conjunction with other resistance genes, may result in a broader spectrum of resistance, posing a significant challenge to the poultry industry. Our results are consistent with previous research on the coexistence of *qnr* and β-lactamase genes (Röderova et al., 2017; Aworh et al., 2023). In addition, we found that the isolates carrying *qnrS1* also frequently harbored *tet(A)*. Previous reports have suggested that ESBL-producing *E.coli* strains are more likely to acquire *mcr-1* (Zhao et al., 2022); however, this was not observed in our study. This discrepancy may be due to our investigation focusing solely on gene identification in ESBL-producing isolates.

To investigate the molecular evolution of bacteria, MLST is essential. Of the 13 STs identified among the 18 isolates, ST1286 (16.67%) was the most common. The distribution of ARGs varied even among isolates sharing the same STs. ST10 has been commonly identified in animals, suggesting that its prevalence may not be restricted by geographical location or animal species (Li et al., 2024; Yuan et al., 2024; Reid et al., 2017). Furthermore, ST10, an extraintestinal pathogenic *E. coli*, has been linked to human infections and warrants greater attention (Manges et al., 2019). In our investigation, each farm yielded a single ST10 strain. O16: H32 was the most commonly identified serogroup in this study. For additional molecular typing, CH typing is utilized to evaluate the clonal diversity of ST subtypes and is also a reliable predictor of MLST profiles (Weissman et al., 2012). A total of 13 CH types were identified. The dominant CH type was FumC174/FimH23. Strains with the same CH types typically congregate within the same serotypes and STs, as evidenced by the observation that certain CH types are associated with particular serotypes and STs. The phylogenetic tree also sheds light on the genetic relationships among the isolates. In general, the clade structure aligned well with the typing results, with the isolates sharing the same STs clustering closely together on the same branches. These isolates were obtained either from the cloacal swabs from the same poultry farm (e.g., 2,021,039 and 2,021,037; 2,021,029, 2,021,030, and 2,021,031), which differed by 161–845 SNPs, or from the cloacal and environmental samples from the same poultry farm (e.g., 2,021,091 and 2,021,056), which differed by 2,134 SNPs. According to reports, hens have the potential to contaminate their surroundings and act as vectors, facilitating the spread of disease to workers and others (Chuppava et al., 2019). Two isolates of ST10 that were collected from separate farms showed high genetic relatedness, with a difference of 938 SNPs. In conclusion, further investigation is needed to determine whether cross-contamination between the environment and poultry has occurred. In any case, prudent use of antibiotics on farms and effective disinfection practices are essential.

## 5 Conclusion

This study investigated the incidence, resistance profiles, and genetic traits of diarrheagenic *E. coli* in Shanghai, China. MDR *E. coli* was found to be fairly severe, and ARGs were widely distributed among ESBL-producing *E. coli* strains. The isolates with same STs exhibited very close genetic relationships based on the SNP phylogenetic tree, highlighting the need for enhanced surveillance to prevent further transmission. Nevertheless, it should be noted that this study did not include WGS of all isolates to identify specific resistance genes and mechanisms. Therefore, future studies should focus on investigating the transmission dynamics between isolates and human samples to gain a better understanding.

## Data Availability

The original contributions presented in the study are included in the article/Supplementary material, further inquiries can be directed to the corresponding author.

## Footnotes

^1^ http://www.genomicepidemiology.org/
